# An Altered Treatment Plan Based on Direct to Consumer (DTC) Genetic Testing: Personalized Medicine from the Patient/Pin-cushion Perspective

**DOI:** 10.3390/jpm2040192

**Published:** 2012-10-30

**Authors:** Jessica D. Tenenbaum, Andra James, Kristin Paulyson-Nuñez

**Affiliations:** 1 Duke Translational Medicine Institute, Durham, NC 27715, USA; 2 Department of Obstetrics & Gynecology, University of Virginia, Charlottesville, VA 22908, USA; E-Mail: andra.james@virginia.edu; 3 Department of Obstetrics and Gynecology, Duke University School of Medicine, Durham, NC 27705, USA; E-Mail: kristin.nunez@dm.duke.edu

**Keywords:** personalized medicine, direct-to-consumer genetic testing, genetic counseling, thrombophilia, venous thromboembolism, clotting disorders, pregnancy

## Abstract

Direct to consumer (DTC) genomic services facilitate the *personalized* and *participatory* aspects of “P4” medicine, but raise questions regarding use of genomic data in providing *predictive* and *preventive* healthcare. We illustrate the issues involved by describing a pregnancy management case in which a treatment plan was modified based on a DTC result. A woman whose personal and family history were otherwise unremarkable for thromboembolism learned through DTC testing about the presence of a prothrombin (factor 2) gene mutation (rs1799963). Twice daily injections of enoxaparin were recommended throughout pregnancy for this patient who, without prior knowledge of this mutation, would not have been offered such therapy. Moreover, genetically based medical guidelines are a moving target, and treatment of thrombophilic conditions in asymptomatic patients is controversial. We address the state of the art in actionable personalized medicine with respect to clotting disorders in pregnancy, as well as other factors at play— economics, patient preference, and clinical decision support. We also discuss what steps are needed to increase the utility of genomic data in personalized medicine by collecting information and converting it into actionable knowledge.

## 1. Introduction

Much has been written in recent years, both in biomedical literature and in the popular media about the concept of “P4” medicine and each of the ‘P’s respectively: predictive, preventive, personalized, and participatory [1,2,3,4,5]. The general idea behind P4 medicine (and a number of similar, partially overlapping concepts—genomic medicine, stratified medicine, precision medicine, individualized medicine, *etc.* [6,7,8,9]) is that medicine should not be practiced in a reactive, one-size-fits-all approach. Interventions, both therapeutic and preventive, should instead be targeted for the individual patient based on his or her specific circumstances—genotype, environment, lifestyle, *etc.* The emergence of direct-to-consumer (DTC) genetic testing represents a significant achievement for realizing the P4 vision, particularly with respect to medicine that is both personalized and participatory. A person’s genetic information is, by definition, personalized. It represents a DNA sequence that is unique to each person. By taking the initiative to obtain (and pay for) genomic data, a person assumes a proactive stance, changing from a passive recipient of health care services to an active player in the management of one’s own health and well-being. With respect to prediction and prevention, however, integration of genomic data into medical practice continues to fall short. Likewise, consumers have a limited understanding of the methodologies involved with testing and what those results may actually mean to their health management. Hundreds of genetic variants have been linked to a given disease, symptom, or syndrome [10]. However, in most cases, these variants are unlikely to have clinical manifestations. They have small risk ratios, barely significant p-values, or no actionable recourse. Even when a recommended course of action exists, often it is a restatement of commonly accepted guidelines for good health, e.g., maintain a healthy body mass index, exercise, and refrain from smoking [11].

We present a real-world example of an altered treatment plan based on a DTC genomic test result from both the health care provider’s and the patient’s perspective. We also discuss the relatively immature and fluid state of evidence-based genomic medicine in the context of clotting disorders, other facets of individualized therapy, and what steps are needed to convert genomic information into actionable knowledge.

## 2. P4 Medicine: A Real World Example

### 2.1. Case Presentation

A 33 year-old woman electively underwent DTC testing in the context of exploring a potential scientific collaboration. The patient’s area of professional expertise is in the field of bioinformatics as it relates to personalized medicine, thus she was relatively well-versed with DTC technologies and genomic data, both the capabilities they offer and their current limitations. The service was performed as a professional courtesy. The patient’s specific results revealed that she was heterozygous for the common prothrombin gene mutation (rs1799963), also known as factor 2. She had no personal or family history of venous thromboembolism (VTE), recurrent pregnancy loss or poor pregnancy outcome (defined as fetal growth restriction, placental abruption, preeclampsia or stillbirth). In 2009, after 14 months of infertility, the patient was referred to a reproductive endocrinology and infertility clinic where she was evaluated for unexplained infertility and, ultimately, conceived twins with the use of fertility medication. Though she did not think it was terribly important, the patient informed her fertility specialist about her prothrombin gene mutation status. The specialist felt this information was significant and that the patient’s specific genotype would modify her pregnancy management to include 30 mg twice daily of enoxaparin and 81 mg of aspirin daily through the first 12 weeks of a pregnancy. She was also referred to a high-risk obstetrician for the remainder of the pregnancy. Although the DTC testing was performed in a CLIA (Clinical Laboratory Improvement Amendments) certified lab, the specific genotype was confirmed through a physician-ordered test prior to commencing prophylaxis. At 12 weeks gestation, the patient was seen in the high-risk obstetrical clinic by a maternal-fetal medicine physician who specialized in clotting disorders (AJ) to discuss pregnancy management and delivery in light of her heterozygous status. At that time, the specialist recommended decreasing her enoxaparin to 40 mg daily through 28 weeks gestation at which time she would be converted to 40 mg twice daily and begin monthly ultrasounds followed by non-stress tests at 28 weeks gestation. Finally, pneumatic compression devices were recommended in labor along with 40 mg enoxaparin daily starting twelve hours after delivery and continuing through six weeks postpartum.

The patient’s pregnancy was uncomplicated through 29 weeks gestation when a short cervical length was noted on ultrasound. At approximately 30 weeks gestation, the patient had premature rupture of membranes and premature labor. Due to fetal positioning, an emergent cesarean section was performed. Both babies were delivered without complication and were discharged following a short stay in the newborn intensive care unit. Now two years old, they are reported to be developmentally normal and healthy. Notably, the patient's complications were likely related to her twin gestation and not due to her thrombophilia genotype. In addition, the patient continued her use of enoxaparin through five weeks postpartum and experienced no venothromboembolitic events.

### 2.2. Relative Risk Calculation

**Figure 1:**
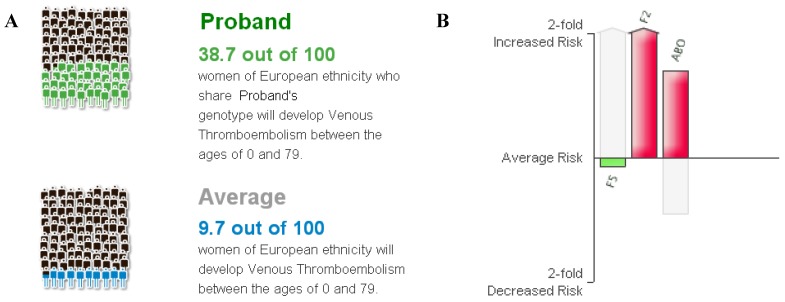
Graphical representation of probability and genetic risk factors. (© 23andMe, Inc. 2007-2012. All rights reserved; distributed pursuant to a Limited License from 23andMe.) (**A**) Colored figures represent the number of people on average out of one hundred who are likely to develop venous thromboembolism (VTE) over the course of a lifetime. Green figures represent the patient’s personal reported risk; blue figures represent the average risk for females of European descent; (**B**) The patient’s relative risk for each of 3 reported markers. Her factor V genotype (CC) is reported to confer a .95 odds ratio; factor 2 genotype (AG) odds ratio = 3.8×; ABO genotype (CC) odds ratio = 1.62×. (These values are displayed on the website when the user hovers the mouse over the colored bars.)

The patient’s specific *reported* risk for VTE has changed over time since the original results were provided through the DTC company’s website. The original predicted risk was based on two variants, prothrombin gene mutation and factor V Leiden, and did not take gender into account. The original lifetime risk was determined to be approximately 55%, roughly twice the risk of the rest of the population (24.7%) [12]. Starting in July, 2009, the DTC report began to include sex-specific incidence rates that in addition to distinguishing between men and women, appeared to be lower than the previous incidence data [12]. Using this new data resulted in a lifetime risk estimate of 28.1% (*vs*. the average of 9.7%) for a woman carrying one copy of the prothrombin gene mutation. This was the most up-to-date figure when the patient learned she was pregnant. In March, 2012, the company added the ABO polymorphism to the risk calculation, resulting in a lifetime reported risk estimate of 39.7%, approximately 4 times the average risk of females of European ancestry (9.7%) [12]. The graphical representation of this information provided on the DTC company’s website can be seen in Figure 1.

### 2.3. Clinical Perspective

The prothrombin gene mutation is present in approximately 2% of the general population and is most prevalent (3%) in southern Europeans [13]*.* OMIM (the Online Mendelian Inheritance in Man) cites a 2001 study by Pihusch *et al.* in which recurrent spontaneous abortions in the first trimester were associated with heterozygosity for the prothrombin gene mutation. The author suggested that because thrombin is known to activate tissue components represented in the placenta, increased prothrombin levels might influence placental function [14]. A 2002 review found evidence in the literature for a link between acquired thrombophilias and adverse outcomes in pregnancy; however they cautioned that the studies that had been published to date were too small to draw confident conclusions. They suggested that screening should not become part of routine clinical practice until there was more evidence to suggest that thromboprophylaxis during pregnancy improves outcome [15]. In a 2006 review of 81 studies, 72 of which involved pregnancy, the authors stated that “Significant risks for VTE and adverse pregnancy outcomes have been established with individual thrombophilic defects” [16]. Finally, a 2010 study of 2,034 nulliparous women found that carriers of the prothrombin gene mutation carried an odds ratio of 3.58 for adverse pregnancy outcomes, but that the majority of asymptomatic carriers of thrombophilia mutations had successful pregnancies.

In contrast to the findings described above, a 2010 study performed secondary analysis on a prospective observational cohort. Among the 4,167 low-risk singleton pregnancies included in the analysis, 157 (3.8%) of the women in the study carried the prothrombin gene mutation. No association was found between the mutation and adverse pregnancy outcomes [17], but with 157 affected subjects, the study was underpowered to detect certain adverse pregnancy outcomes. Another study, published in late 2010, looked at the risk for pregnancy-related venous thrombosis in carriers of factor V Leiden and prothrombin mutations [18]. This group looked at more than 600,000 pregnancies in almost 400,000 women at 18 Norwegian hospitals over a period of 14 years. This study determined that 2,015 women would need to be screened, and 157 given thromboprophylaxis, in order to avoid one VTE. They concluded that although inherited thrombophilias do, in fact, increase the risk of VTE during or after pregnancy, the overall risk is sufficiently small that general screening is not warranted. They do not, however, directly address the question of what to do if the patient has been “pre-screened”. 

### 2.4. Patient Perspective

Had the patient not chosen to undergo DTC testing, it is unlikely that she would have ever known about her factor 2 status. With no family history of clotting events nor personal history of poor pregnancy outcome, the patient would not have had reason to check for these mutations. Once known, however, the genotype became one of many factors that the patient and her health care providers considered significant enough to incorporate into her healthcare management. Additional factors to be considered included age, fertility, economics, and, in this case, a strong aversion to needles. As noted above, the patient required fertility medication to conceive, and at the time of pregnancy, was 35 years old. While loss of a wanted pregnancy is always tragic, it is all the more difficult when one feels that there will be limited opportunities to try again. The patient’s financial situation was such that the majority of the cost of the enoxaparin was covered by insurance, and the approximately $50 prescription co-payment was not prohibitive. Of far greater concern to this particular individual was the prospect of having to self-administer a medication twice daily via injection into the fatty tissue around the abdomen. To her, compliance with the recommended treatment felt like an extreme precautionary measure. It required the patient to overcome a stronger-than-average lifelong aversion to needles, but was ultimately tolerated because of the perceived risk of complications due to her genotype, as well as her desire to have a family.

As noted above, the patient’s decision to undergo DTC testing was motivated by professional considerations and the test was performed free of charge. The cost of the service at the time was approximately $500 and the patient did not feel she could justify the purchase at that price point. Even at the August, 2012 price of $299, the patient believes it unlikely she would have elected to pay for the service out of pocket. However, when the company ran a promotion that included a kit for $99 with a commitment to pay a $5 monthly fee for one year (total commitment of $159), she did purchase a kit as a gift for her father. Cost, therefore, is a relevant factor to consider with regards to future DTC testing motivation and penetrance. As genomic technology continues to advance, and the price of genotyping continues to fall, these services will increasingly be economically within reach for a larger segment of the population. With that said, there remains the risk that elective DTC testing could further exacerbate existing healthcare disparities between different socioeconomic groups.

### 2.5. Prediction, Prevention, and Probability

The era of P4 medicine encourages the empowerment of patients as informed consumers. However, it has also been noted that not all patients possess the knowledge and skills required to interpret health information that includes uncertainty and probability values [19]. Based on all of the independent risk factors (multiple gestation, advanced maternal age, prothrombin gene mutation status [20]), the patient was told that she had a 5% chance of an adverse outcome without thromboprophylaxis. To clinicians this probability is relatively high, whereas to her this number seemed relatively low. The magnitude was put into perspective for the patient by another finding from her DTC results from which the blue-eyed patient was surprised to learn that her eye color was reported as “Likely Brown.” In fact, the chances of having blue eyes given the patient’s specific genotype was only 7%. As she described to the genetic counselor, her improbable blue eyes instilled in her mind the notion that “seemingly unlikely things can happen to me.” With this added perspective, a 5% likelihood of an adverse outcome no longer seemed so small. Ultimately, the patient opted to base her decision to use enoxaparin not only on the physician’s recommendation but also, and perhaps more importantly, on her own perception of risk. Even for this patient, an expert in bioinformatics and genomic data, the decision was not straightforward. It seems likely that any confusion and uncertainty would only be compounded for a less well-versed layperson.

## 3. Discussion

One fundamental question in this case (the fundamental question from the patient’s perspective) is: what is the right course of action? As is apparent from the fluctuating relative risk calculation in Section 2.2 above, it is not (yet) possible to state definitively what the right treatment may be. At best, we can posit the optimal course of action based on the information we possess at a given point in time. Although working groups such as American College of Medical Genetics and Evaluation of Genomic Applications in Practice and Prevention (EGAPP) have provided consensus guidelines to assist with determining who should be offered testing among asymptomatic individuals [21,22], DTC testing is driven by patient request and the company’s need to offer, perhaps, a greater panel of markers in order to be competitive with the market. In addition, findings and guidelines focus primarily on screening. Less information is available regarding what to do in the case of a patient with inherited thrombophilia but no other known risk factors, perhaps because this situation arose rarely before the advent of DTC testing. As the option to genotype becomes more common and economically accessible to the public, presumably more carriers of thrombophilic mutations will know their own genotype. 

From a strictly economic perspective, if the cost of the intervention exceeds the product of the probability of such an event and the cost of medical treatment for adverse events, then preventive care would not appear to be the appropriate action. Reality, of course, is more complex. Thromboprophylaxis does not reduce the probability of an adverse event to zero, and there are a variety of possible adverse outcomes to consider. While a miscarriage or pregnancy complication may be devastating, a lethal clotting event precludes additional pregnancy attempts. In addition, should the patient’s age and fertility status be taken into account? These questions are unlikely to be resolved in the near term. Rather, they are matters for debate, by society and by experts in health care, ethics, policy, and law. Currently, there is general consensus that in individuals without a history of VTE, screening for thrombophilia not cost-effective [23]. However, the utility of anti-clotting prophylaxis for patients with inherited thrombophilia but no other risk factors remains controversial. 

The case presented here is a real-world example that hints at the promise of DTC testing and P4 medicine, but also demonstrates how far we have yet to go. As described above, the scenario is both personalized and participatory. However, even in the studies that have shown a link between the prothrombin mutation and poor pregnancy outcome or VTE, this link falls far short of being *predictive*. The vast majority of the 1 in 50 people with this mutation suffer no negative consequences, and will never become aware of their at-risk status. Moreover, there is insufficient evidence to date to conclude that thromboprophylaxis during pregnancy actually improves outcomes. Until appropriately powered randomized controlled trials of this treatment can be performed, the knowledge gained from DTC testing for heterozygous prothrombin mutations will continue to fall short of enabling truly *preventive* medicine.

### Future Steps Needed

For clotting disorder management in pregnancy, as with many other aspects of medicine (personalized or otherwise), what is most needed is evidence. Particularly for genomic medicine, in which the number of parameters increases exponentially, it is all the more crucial to have large numbers of subjects for well-powered studies. In addition, as genomic data is incorporated into medical decision making, the sheer number of variables that a clinician must take into account will rapidly exceed the capabilities of human cognition [24]. For this reason, appropriate information technology will be critical, playing a key role both for gathering data at the point of care and providing decision support to clinicians. These tools will need to be seamlessly integrated with both the technology and the workflow around the electronic health record. With increased adoption of electronic health record systems, every person who comes into contact with a health care provider becomes a valuable contributor to our accumulating body of evidence. In this way, we can enable a “learning healthcare system” that functions to both generate and apply data to improve medical care and human health [25].

One of many ways in which this patient’s circumstances were unusual was the fact that she lived within 30 min of a tertiary care facility with an abundance of specialists, including experts in coagulation disorders in pregnancy. Her pregnancy management plan was thus informed by the most recent guidelines and discoveries in the field. The situation for a pregnant woman in rural America would likely be quite different. Even a general high-risk obstetrician might be more than a hundred miles away. In this scenario, it is all the more urgent that technology provide timely, up-to-date, user-friendly decision support in non-standard cases. It is also critical that small, rural practices embrace and adopt these technologies.

## 4. Conclusions

The case we describe above highlights a number of issues and challenges around DTC testing and genomic medicine. And we note that these issues arose in the context of highly specialized experts in a tertiary care environment and a well-informed and highly educated patient. While the case brings to light many as yet unanswered questions, we remain optimistic about the promise of personalized medicine for the future. Today we only recognize a small subset of the polymorphisms at play, but that knowledge will continue to increase over time. As it does, DTC genetic testing and other novel technologies to ascertain previously inaccessible information should and will serve to improve prospects for delivery of P4 medicine that is truly predictive and preventive. 
